# Revisiting the Global Overfat Pandemic

**DOI:** 10.3389/fpubh.2020.00051

**Published:** 2020-02-25

**Authors:** Philip B. Maffetone, Paul B. Laursen

**Affiliations:** ^1^Independent Researcher, Ormond Beach, FL, United States; ^2^Auckland University of Technology, Auckland, New Zealand

**Keywords:** overweight, obesity, cardiometabolic, chronic disease, healthcare costs

## Abstract

The previously described overfat pandemic, estimated to be 62–76% worldwide, is comprised of individuals with excess body fat sufficient to impair health. The overfat condition is common in those who are overweight and obese, and can also occur in significant numbers of normal-weight non-obese individuals. Being overfat increases the risk for a wide spectrum of common cardiovascular and metabolic (cardiometabolic) abnormalities, chronic diseases and physical impairment. In some ethnicities, up to 40% or more of those who are normal-weight and non-obese may be overfat, a figure twice that used in the original global overfat estimates. In addition to the rates of overfat outpacing overweight and obesity, non-White populations outnumber Whites 6:1, with the recently estimated overfat prevalence being low when considering ethnicities such as Asians, Chinese, Africans and Latin Americans, including these individuals living among predominantly White populations. An awareness of the extent of the overfat pandemic is important because excess body fat can precede cardiometabolic risk factors, chronic diseases, and physical disabilities, and can reduce quality of life and increase healthcare expenditure. The purpose of this Perspective is to demonstrate that the global overfat prevalence of 62–76% may be considerably underestimated.

## Introduction

The term *overfat* was defined as excess body fat that impairs health (1). The problem can occur in those who are overweight, obese, and include significant numbers of normal-weight non-obese individuals (2, 3). Excess body fat can contribute to cardiovascular and metabolic (cardiometabolic) health impairment including various risk factors such as abnormal blood glucose, high-density lipoprotein cholesterol (HDL), triglycerides and blood pressure, and progress to a variety of diseases including type 2 diabetes, non-alcoholic fatty liver, cancers, Alzheimer's and cardiovascular diseases (4–6), including physical impairments (7). As such, the overfat pandemic has created a major global economic burden (8, 9).

As a subcategory of overfat, measured by waist circumference, the prevalence of abdominal obesity, which poses a greater cardiometabolic risk than excess fat elsewhere in the body, is increasing dramatically worldwide (10–12). The increasing number of people who are normal-weight and non-obese but overfat was first described by Ruderman et al. (2) as *metabolically-obese, normal weight* individuals (2, 13), with the overfat prevalence in this population estimated at ~20%. More recently, Gujral et al., referred to the problem as *metabolic abnormality but normal weight* (MAN), showing a wide variety in the overfat prevalence between various ethnicities, with excess body fat in normal-weight non-obese individuals exceeding 40% in some ethnic groups (3). In a US population, MAN was defined as a BMI of 18.5 to 24.9 kg/m^2^ for White, African American, and Hispanics or a BMI of 18.5 to 22.9 kg/m^2^ for South Asian and Chinese Americans, along with ≥2 cardiometabolic abnormalities.

As a surrogate measure of body fat, the body mass index (BMI) can misclassify many individuals who are normal-weight and non-obese, but overfat, and therefore miss an opportunity to prevent or treat excess body fat and its associated cardiometabolic and physical impairments. (14, 15). In the U.S., data shows that a high percent body fat can exist even in lower BMI categories (16). One reason is ethnicity, where a BMI cut point of 30 kg/m^2^ in Whites is equivalent to lower BMI levels for other ethnicities including South Asians, Chinese Americans, and African Americans with cardiometabolic impairment (3, 17, 18). BMI fails to consider whole body fat distribution, in particular abdominal fat, which is associated with excess around the heart, liver and kidneys, that can be more pathogenic than general obesity (19). While BMI is a screening tool for overweight and obesity, it has limitations. Many researchers and clinicians regularly use it, with continued recommendations made by healthcare organizations such as the U.S. Preventive Services Task Force (20).

Instead of BMI, the waist-to-height ratio (WHtR), an indirect measure of body fat, is more strongly related to excess body fat and cardiometabolic impairment, and is an easier measurement to obtain (21, 22). Epidemiologic studies demonstrate that increased abdominal fat assessed through WHtR can predict adiposity-related risk, although, like BMI, it does not accurately estimate body fat percentage (15). WHtR may be the most convenient, inexpensive, and valuable clinical indicator of health and overfat risk for use in all ethnic groups of adults and children, with a simple recommendation that the waist should be less than half of a person's height (15, 22–25). Unfortunately, the various methods for measuring body fat, including bioelectrical impedance, dual-energy X-ray absorptiometry (DXA), waist and WHtR, BMI, and others, makes comparison across studies difficult, contributing to the difficulty of estimating overfat prevalence.

The overfat condition can have an inverse relationship with physical activity, as increased body fat can develop despite increased rates of exercise (26). This paradox appears to include the general population, and is also evident in athletes (27, 28) and the military (29).

Global populations share a common risk factor—the increased consumption of processed carbohydrates, especially sugar, which may be the largest contributing lifestyle factor to the overfat pandemic (30). Sugar-sweetened beverages are the single largest source of added sugar and the top source of energy intake in the U.S. diet (31–33). However, their increased consumption in developing nations is significant too (34), with sugar-sweetened beverages strongly associated with chronic illness (35). In India, for example, annual sugar sweetened beverage consumption increased from 2 L per capita in 2000 to over 11 liters per capita in 2013 (36, 37).

While the global overfat prevalence was previously estimated to between 62 and 76%, this may have been considerably underestimated due to a number of factors discussed in this perspective.

## Rising Rates of Overfat

Since the first estimation of global overfat prevalence was written in 2016 (published in 2017) (1, 38), a number of factors appear to indicate this estimation is low. In addition to emerging evidence regarding overfat outpacing overweight and obesity noted above, it includes the significantly higher percentage of normal-weight non-obese non-White overfat populations, especially in the two most populated regions, South Asia and China.

### Ethnicities

While studies demonstrating ethnic differences in body fat content with differences in BMI cut-offs have appeared for some time (reviewed by Shah (18), a growing consensus shows there is a much higher rate of excess body fat in normal-weight non-obese non-White individuals. Compared with Whites, Asians, Hispanics, and Blacks may have a significantly higher overfat prevalence, which is not explained by factors such as demographics and behavior (3). These studies help confirm previous works that a BMI cut point of 30 kg/m^2^ in Whites was equivalent to lower BMI cut points for South Asians, Chinese, Blacks, and Aboriginal Canadians in terms of body fat, cardiometabolic impairment and chronic disease (17, 18, 39, 40).

Reasons for such significant growth of overfat populations in developing areas could be multifactorial, including genetics and lifestyle. The *adiposity rebound* is a condition of low weight and thinness at 1 to 2 years of age, often accompanied by low birth weights, developing into excess body fat and impaired glucose tolerance and diabetes in adulthood (41), a phenomenon also demonstrated in individuals growing up in post-World War II Finland (42). The *nutritional transition* occurring in developing populations in Asia and Africa may also have impacted the overfat prevalence. As the second most primarily non-White populated continent behind Asia's 4.5 billion people, Africa's population of 1.2 billion also shares a growing common cardiometabolic risk factor: overfat.

While non-Whites outnumber Whites significantly worldwide by ~6:1, in many developed nations that are primarily White, there are also modest numbers of other ethnicities at high risk of being overfat. For example, in a U.S. population, Gujral et al. (3) showed that, like their counterparts in developing countries, significantly higher percentages of normal-weight non-obese overfat individuals occurs in Asians, Hispanics and Blacks compared to Whites (see Table 1). This trend was also previously shown in South Asians who move to Western countries (43). The United Nations estimates that there are over 370 million aboriginal, native or indigenous people worldwide.

**Table 1:** Percentage of combined cardiometabolic abnormalities and excess body fat in normal-weight non-obese mixed U.S. population [from Gujral et al. (3)].

South Asian	43.6%
Hispanic	38.5%
Chinese	32.2%
Black	31.1%
White	21.0%

### South Asia

India, Pakistan, Bangladesh, Bhutan and other South Asian countries have a significant and increasing prevalence of excess body fat, cardiometabolic impairment, and high burden of disease associated with insulin resistance and diabetes, hypertension, hypertriglyceremia and low serum levels of high density lipoprotein (HDL) (44, 45). For the same BMI, body fat is 7–8% higher for Indians compared to Whites (46). South Asia also has a population of ~1.92 billion people, with the highest percentage of overfat individuals in the normal-weight non-obese category, recently estimated at 44 and 41% (3, 47).

### China

China was considered the largest overweight population in the world in 2014 with about 26% of adults representing about 364 million people (48). However, a recent study showed the prevalence of overfat Chinese adolescents from bioelectrical impedance to be 36% in girls and 24% in boys (49). These overfat adolescents can become overfat adults with increased risk of chronic disease and physical impairment (50). In adults, the age-adjusted prevalence of abdominal obesity measured by waist circumference was 52% in men and 35% in women in 2011, increasing dramatically from 17 to 39% between 1997 and 2009 (51, 52). Like other developing nations, China's leading cause of death is now chronic disease, particularly cardiometabolic impairment, having surpassed infectious diseases (53). Hypertension, for example, is not uncommon; using the recent definition of hypertension being blood pressure >130/80 mm Hg would estimate that 267 million Chinese in the 45–75 age group would be hypertensive (54). Over a 5-year period between 2002 and 2007, the prevalence of one or more cardiometabolic abnormalities in normoweight Taiwanese people (39% male) 16 to 45 y significantly increased from ~30 to ~40% (55).

### The Continuing Overfat Trend

In addition to the increasing prevalence of overfat populations in Asia and Africa, developed nations continue leading the way. In the US, for example, between 2013 and 2016 the estimated overfat prevalence in adults increased from 87% (38, 56) to 91% with the estimated prevalence of overfat U.S. children increasing from 52 to 69% (26, 57). In addition, developed populations have modest numbers of non-Whites with significant numbers of normal-weight non-obese ethnicities who are overfat (Table 1).

Considering these factors, especially higher percentages of overfat in normal-weight non-obese non-White populations, overfat rates may be outpacing more modest increases in overweight and obesity (56–58).

## Discussion

The next generation of obesity research may focus less on overweight and obesity and more on body fat, as the latter is more relevant to the development of cardiometabolic risk factors, chronic disease, and physical impairment; all significant sources of rising healthcare costs in both developed and developing nations. Rather than relying on BMI, measurements of body fat through direct or indirect methods, such as DXA or WHtR, respectively, can provide valuable data for researchers and clinicians. For home use, WHtR is a simple and accurate method for individuals to help manage health.

Previous estimates of global overfat prevalence were primarily based on overweight and obesity rates, plus ~20% of normal-weight non-obese individuals. As updated research reveals significant overfat conditions in non-White ethnicities at lower BMI ranges, especially in large populated areas such as Asia and Africa, and considering that the world population of non-Whites is about six times greater than Whites (see Table 2), the recent global overfat prevalence of between 62 and 76% may have been significantly underestimated.

**Table 2:** Estimated non-white populations in billions in selected regions of the world (Global population of whites account for <1 billion; estimated world population of 7.7 billion).

South Asia	1.92
China	1.44
Africa	1.22
Latin America	0.64
Total	5.22

Estimating the number of overfat people in the world is important because excess body fat can significantly affect quality of life, precede chronic disease and physical disability, and dramatically increase healthcare expenditure. A variety of lifestyle issues, including dietary, environmental, exercise, stress, along with epigenetic factors, must be considered in the assessment, treatment and prevention of this condition.

Even defined within the discussion of this paper, there is further risk of missing many people who are in the early stages of being overfat as they may not have obvious disease risk, i.e., one cardiometabolic risk factor. This is another missed opportunity for prevention, best implemented sooner rather than waiting for more obvious excess fat or additional cardiometabolic impairment. As such, a modified definition of overfat was previously presented and emphasized here: excess adiposity indicted by direct (DXA) or indirect (WHtR) measures combined with at least one additional measurable risk factor of impaired cardiometabolic or physical health (26). Universal public health guidelines addressing the overfat pandemic will require far more emphasis on reducing the consumption of refined carbohydrates, including added sugars (25), with taxation being one effective intervention (59).

As important as making overfat estimations, both public health officials and clinicians worldwide have sufficient, cost-effective assessment and treatment tools available to reverse this preventable condition, and the overfat pandemic.

## Conclusion

Case definitions for overweight and obesity have long been developed and researched, with the awareness that excess body fat poses significant health risks to all individuals. As previously described, excess body fat that impairs health is defined as overfat. The total prevalence of overfat equals those who are overweight, plus those who are obese, plus normal-weight non-obese individuals with excess body fat. Future studies associated with overweight and obesity should also consider the overfat condition.

Determining the global overfat prevalence is not the purpose of this paper, but is instead to demonstrate that the recent estimation of 62–76% may have been significantly underestimated. This makes the overfat pandemic a serious condition requiring interventions to address the problem by governments, healthcare agencies and clinicians, including education of the public.

## Author Contributions

PM wrote the initial manuscript. PL reviewed and edited the manuscript.

### Conflict of Interest

The authors declare that the research was conducted in the absence of any commercial or financial relationships that could be construed as a potential conflict of interest.
